# CNN-based prediction using early post-radiotherapy MRI as a proxy for toxicity in the murine head and neck

**DOI:** 10.2340/1651-226X.2025.44020

**Published:** 2025-09-25

**Authors:** Bao Ngoc Huynh, Manish Kakar, Olga Zlygosteva, Inga Solgård Juvkam, Nina Edin, Oliver Tomic, Cecilia Marie Futsaether, Eirik Malinen

**Affiliations:** a Department of Medical Physics, Oslo University Hospital, Oslo, Norway; b Department of Radiation Biology, Institute for Cancer Research, The Norwegian Radium Hospital, Oslo University Hospital, Oslo, Norway; c Department of Physics, University of Oslo, Oslo, Norway; d Institute for Oral Biology, Faculty of Dentistry, University of Oslo, Oslo, Norway; e Faculty of Science and Technology, Norwegian University of Life Sciences, Ås, Norway

**Keywords:** head and neck, mice, radiotherapy, deep learning, convolutional neural network, toxicity early detection, magnetic resonance imaging

## Abstract

**Background and purpose:**

Radiotherapy (RT) of head and neck cancer can cause severe toxicities. Early identification of individuals at risk could enable personalized treatment. This study evaluated whether convolutional neural networks (CNNs) applied to Magnetic Resonance (MR) images acquired early after irradiation can predict radiation-induced tissue changes associated with toxicity in mice.

**Patient/material and methods:**

Twenty-nine C57BL/6JRj mice were included (irradiated: *n* = 14; control: *n* = 15). Irradiated mice received 65 Gy of fractionated RT to the oral cavity, swallowing muscles and salivary glands. T2-weighted MR images were acquired 3–5 days post-irradiation. CNN models (VGG, MobileNet, ResNet, EfficientNet) were trained to classify sagittal slices as irradiated or control (*n* = 586 slices). Predicted class probabilities were correlated with five toxicity endpoints assessed 8–105 days post-irradiation. Model explainability was assessed with VarGrad heatmaps, to verify that predictions relied on clinically relevant image regions.

**Results:**

The best-performing model (EfficientNet B3) achieved 83% slice-level accuracy (ACC) and correctly classified 28 of 29 mice. Higher predicted probabilities of the irradiated class were strongly associated with oral mucositis, dermatitis, reduced saliva production, late submandibular gland fibrosis and atrophy of salivary gland acinar cells. Explainability heatmaps confirmed that CNNs focused on irradiated regions.

**Interpretation:**

The high CNN classification ACC, the regions highlighted by the explainability analysis and the strong correlations between model predictions and toxicity suggest that CNNs, together with post-irradiation magnetic resonance imaging, may identify individuals at risk of developing toxicity.

## Introduction

Radiotherapy (RT) of head and neck cancer (HNC) patients can lead to acute and chronic toxicities, including radiation dermatitis, oral mucositis, dysphagia, hyposalivation, xerostomia, pain and weight loss [1–3], which can cause discomfort and inconvenience reducing patient quality of life [1]. Severe toxicities during RT can impair patient tolerance, leading to treatment delays or dose reductions and potentially compromising RT efficacy [3]. Therefore, early toxicity prediction, especially before symptoms worsen or become pronounced, is essential for personalized care and symptom management throughout RT [3, 4].

The growing availability of clinical data provides opportunities for developing machine learning models to identify HNC patients at higher risk of radiation-induced toxicity [4, 5]. Clinical characteristics, such as age and nutritional status, can influence toxicity risk [4, 6]. Tumor-specific factors like location, stage and histology also contribute to risk stratification [6]. Dosimetric parameters, such as planned radiation dose, dose maps and dose-volume histograms (DVH) can also be integrated into models to improve their predictive performance [4, 7, 8].

Medical imaging, including computed tomography (CT), magnetic resonance imaging (MRI) and positron emission tomography (PET), provide anatomical and functional features that may support toxicity risk assessment [5, 8, 9]. These features can be extracted using radiomics [10, 11], which usually requires manual labeling and feature reduction. In contrast, convolutional neural network (CNN) models can analyze medical images directly and potentially capture subtle tissue characteristics relevant for predicting treatment-related toxicity [12]. Despite their strong predictive power, CNNs are often seen as "black boxes" due to limited interpretability [13]. Explainability methods address this by highlighting regions most influential for predictions [14]. In this context, explainability serves as a sanity check: if the model focuses on clinically relevant areas, its predictions are more likely meaningful, whereas reliance on irrelevant regions may indicate spurious associations.

While clinical data from human patients are ideal for developing toxicity prediction models, preclinical studies using laboratory animals offer a controlled environment and allow experimental manipulations unfeasible in human studies [15]. Animal studies are important for testing hypotheses about early biological or imaging biomarkers of toxicity and for developing and validating modelling approaches before clinical translation [16, 17]. Furthermore, while numerous predictive tools are being explored for possible prognostic value in HNC [18], there is a scarcity of approved markers. Integrating CNNs with explainability methods may offer insights into regions affected by toxicity and holds promise for identifying novel imaging-based markers.

This study aimed to evaluate whether CNNs can detect early signs of radiation-induced tissue damage in mouse normal tissue using T2-weighted Magnetic Resonance (MR) images acquired 3–5 days post-irradiation. We hypothesized that CNN models could capture subtle image features associated with radiation-induced tissue changes. To test this, we compared multiple CNN architectures for their ability to distinguish control from irradiated mice, examined correlations between model predictions and five toxicity endpoints and applied explainability analysis as a model sanity check to identify image regions most influential for classification.

## Patients/material and methods

### Animal handling and datasets

Nine-week-old female C57BL/6JRj mice (Janvier, France) were housed under pathogen-free conditions on a 12-hour light/dark cycle, with unrestricted access to standard commercial fodder and water. Standard housing included nesting material and refuge. Mice were 12 weeks old at the start of the experiments.

The present study included 29 mice, which were randomly assigned to a control group (*n* = 15) and an irradiated group (*n* = 14). Mice in the irradiated group received a total dose of 65 Gy in 10 fractions (two fractions per day or one fraction per day), targeting the oral cavity, swallowing muscles and salivary glands. The control mice were treated identically but were not irradiated. The early toxicity endpoints, oral mucositis and lip dermatitis scores, were acquired for all mice (*n* = 29) in the acute phase 8–35 days after irradiation [19, 20]. Three late toxicity endpoints acquired 105 days after radiation were available for subsets of mice [21, 22]. These were saliva production (µl in 15 minutes, *n* = 16), and the percentage areas of submandibular gland fibrosis (*n* = 8) and atrophy of salivary gland acinar cells (*n* = 16).

**Figure 1:**
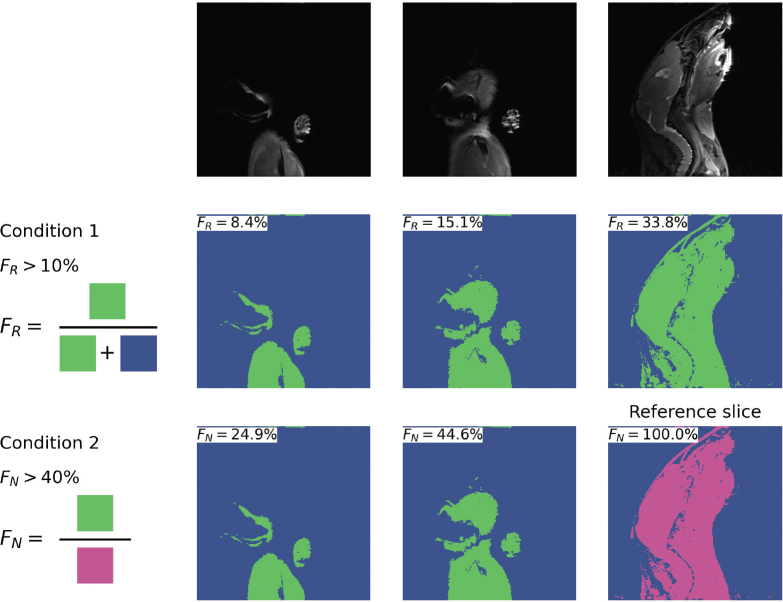
Example results of Huang’s thresholding applied to normalized MR slices (top row), along with the two criteria used for selecting 2D MR slices for CNN model development (middle and bottom rows). The left slice, which did not meet either criterion, was excluded from the dataset. In contrast, the center and right slices satisfied both criteria and were included for model training. Blue and green regions show the background and foreground, respectively. The magenta region depicts the largest foreground area belonging to the reference slice of this particular mouse. MR: Magnetic Resonance; CNN: convolutional neural networks.

This study conforms with the ARRIVE guidelines [23]. Experimental details are given in [19–22]. T2-weighted (T2w) MR images of the head and neck region were obtained from all mice 3–5 days post-irradiation. MR images from each mouse contain 30 sagittal slices of 256 × 256 pixels (0.12 × 0.12 mm^2^, sagittal slice thickness of 0.70 mm). These images were used in a previous study to assess Vision Transformers for image classification [17]. The workflow used for CNN model development including dataset preparation, model training and analysis, is shown in Supplementary Figure B1. Due to the limited sample size (*n* = 29), CNN models were trained using 2D MR slices rather than full 3D MR image series to increase the number of training samples. The 3D MR image series of each mouse was first normalized based on the 0.001–0.999 quantile, slightly improving their contrast, reducing noise and removing outlier voxel values. Thereafter, Huang’s thresholding algorithm [24, 25] was used to identify the foreground (region corresponding to the mouse) and background. Since some MR slices lacked sufficient anatomical information, such as those where the mouse head was partially visible or absent (Figure 1, first row), the following inclusion criteria were applied to select relevant sagittal images:

1. The area of the foreground must cover more than 10% of the sagittal slice under consideration (Figure 1, middle row, *F_R_* > 10%) AND
2. The foreground area of the slice under consideration must be at least 40% as large as the foreground area of the *reference* slice, defined as the sagittal slice with the largest foreground area in the 3D MR image series for that mouse (Figure 1, last row, *F_N_* > 10%).

The selected sagittal MR slices, comprising 304 slices from control mice and 282 slices from irradiated mice, were used to create the dataset for developing CNN models for classifying MR slices into irradiated (class 1) or control (class 0) groups (Supplementary Figure B1). Details about the number of slices selected for each mouse are included in Supplementary Table A1.

## CNN modelling workflow

A total of 17 CNN models based on different architectures, namely VGG [26], MobileNet [27], ResNet [28] and EfficientNet [29], were tested to obtain the highest performing model for classifying the selected MR slices into irradiated or control classes. These CNN models provide a predicted class probability (*P_s_*) of the irradiated class ranging between 0 and 1 for each MR slice. All CNN models were pretrained on the ImageNet dataset [30].

The selected MR slices were divided into five folds for nested five-fold cross-validation (CV) [31], ensuring that each fold (containing three control and two to three irradiated mice) was used at least once as either training, validation or test set. This gave 20 runs in the CV scheme (Supplementary Figure B1&B2). Model training for each CNN architecture was performed across these 20 runs, with each MR slice used as test data in four independent runs (see [32] for details). The resulting test predictions were then ensembled by averaging the predicted class probability (*P_s_*) to produce a final out-of-sample prediction *P_s_*per slice. Note that all MR image slices of the same mouse were always included in the same fold, to prevent information leakage.

The performance metrics (Supplementary C) accuracy (ACC), area under the curve (AUC), F1 score (calculated separately for each class) and Matthew’s correlation coefficient (scaled to 0–1; MCC) were calculated based on the model predictions of all slices. The average of these performance metrics, AvgScore, was used to compare CNN models.

The five models with the highest per-slice validation AvgScore were investigated further by calculating the per-mouse predictions and performance metrics. The per-mouse predicted class probability (*P_m_*) was based on the average predicted class probabilities *P* ^¯^*_s_* of all MR slices belonging to that mouse. The per-mouse performance metrics were calculated similarly to the per-slice metrics but based on the per-mouse predicted class probability *P_m_*. The model achieving the highest per-slice test AvgScore of the five top performing models was used for investigating relationships between model prediction probabilities and toxicity endpoints, as well as for model explainability analysis (Supplementary Figure B1).

Variance of Gradients, VarGrad [33], was chosen for explainability analysis due to its superior stability over other gradient-based explainability approaches and its ability to highlight key regions contributing to model predictions [14]. It creates an explainability heatmap by adding controlled noise to the input and analyzes the variance of the resulting saliency maps [33]. The heatmap highlights regions within MR slices that contribute the most to model predictions.

To analyze the explainability heatmaps, VarGrad values were calculated and compared across three different anatomical regions given in the MR images: the irradiated area (mouth and throat, approximated by the red boundary in Supplementary Figure B1), the non-irradiated area (brain and nape, approximated by the blue boundary) and the background. This comparison acted as a sanity check to assess whether model predictions were based on clinically relevant regions aligning with irradiation status rather than spurious image features.

The irradiation field to the mouse head was previously assessed using planar X-ray imaging [17]. Approximate boundaries for the irradiated and non-irradiated regions of the mouse head were determined using a semi-automatic process. First, the rectangular boundaries of these two regions were manually drawn on the reference MR slices, that is slice with the largest foreground area in each 3D MR image (Figure 1). These initial boundaries were then propagated across the sagittal slices by maintaining their horizontal position while dynamically adjusting their vertical extent. The height of the boundaries was reduced in each slice based on the foreground area, which was pre-defined using Huang’s thresholding algorithm.

### Statistical analysis

Spearman’s rank correlation [34] assessed associations between model-predicted probabilities and late toxicity endpoints (saliva volume, fibrosis, atrophic area). Phi-K correlation [35] was used for acute toxicity (mucositis, dermatitis), as these ordinal scores require a method suitable for ordinal–continuous variable relationships. Note that late toxicities were not available for all mice. The non-parametric two-sided Mann–Whitney U test [36] with a significance level of 0.05 was used to assess significant differences in model prediction contributions (i.e. VarGrad values) across anatomical regions within each MR image.

### Technical information

Development and evaluation of CNN models and the generation of the explainability heatmaps (Supplementary Figure B1, steps 4–6) were conducted on the Orion High Performance Computing cluster at the Norwegian University of Life Sciences using an NVIDIA RTX Quadro (48GB) GPU. Image normalization, thresholding and dataset generation were done on a local computer. The complete source code for data preprocessing, dataset generation, model configuration and training and results analysis can be found at https://github.com/huynhngoc/ous-mice.

## Results

### Model performance

Most CNN models achieved per-slice validation ACC over 75%, except for models in the VGG and MobileNet groups. The ResNet101 model (from the ResNet group) and all EfficientNet models achieved per-slice validation ACC over 80% and AUC over 85%. Detailed validation results are shown in Supplementary Table D1.

Tables 1 and 2 show per-slice and per-mouse performance metrics, respectively, of the five models with the highest per-slice validation AvgScore. While the EfficientNet B1 model obtained the highest per-slice validation AvgScore, the EfficientNet B3 model outperformed the four remaining models across all per-slice test metrics on the test set (Table 1), indicating its stronger generalizability with a per-slice ACC of 83% and an AUC of 91%. In the per-mouse test results, EfficientNet B3, EfficientNet S and EfficientNet B6 models achieved comparable performance, each correctly classifying 28 out of 29 mice in the test results (Table 2; each misclassified mouse resulted in an ACC reduction of 1/29 = 3.4%). Note that the misclassified mouse varied across models, indicating that classification errors were not concentrated on a single case.

**Table 1:** Per-slice performance metrics on validation and test sets of the five highest-performing models.

Model	Accuracy	Scaled MCC	AUC	F1 irradiated	F1 control	AvgScore
*Validation*						
EfficientNet B1	**0.823**	**0.827**	0.892	**0.808**	**0.834**	**0.837**
EfficientNet B6	0.818	0.819	**0.898**	0.802	0.827	0.833
EfficientNet B0	0.816	0.818	0.894	0.804	0.820	0.831
EfficientNet S	0.816	0.818	0.890	0.799	0.828	0.830
EfficientNet B3	0.817	0.819	0.883	0.807	0.820	0.829
*Test*						
EfficientNet B1	0.778	0.778	0.870	0.760	0.794	0.796
EfficientNet B6	0.794	0.793	0.886	0.783	0.803	0.812
EfficientNet B0	0.805	0.807	0.888	0.807	0.804	0.822
EfficientNet S	0.783	0.783	0.889	0.771	0.794	0.804
EfficientNet B3	**0.833**	**0.834**	**0.914**	**0.832**	**0.834**	**0.849**

Highest values are highlighted in bold. MCC: Matthew’s correlation coefficient; AUC: area under the curve.

**Table 2:** Per-mouse performance metrics on validation and test sets of the five highest-performing models.

Model	Accuracy	Scaled MCC	AUC	F1 irradiated	F1 control	AvgScore
*Validation*						
EfficientNet B1	0.940	0.946	0.972	0.939	0.939	0.947
EfficientNet B6	**0.957**	**0.961**	**0.994**	**0.946**	**0.961**	**0.964**
EfficientNet B0	0.948	0.954	0.983	0.945	0.946	0.955
EfficientNet S	0.942	0.947	0.983	0.936	0.945	0.951
EfficientNet B3	0.933	0.940	0.989	0.930	0.931	0.945
*Test*						
EfficientNet B1	0.897	0.897	0.981	0.889	0.903	0.913
EfficientNet B6	**0.966**	**0.967**	**1.000**	0.963	**0.968**	**0.973**
EfficientNet B0	0.931	0.935	0.971	0.933	0.929	0.940
EfficientNet S	**0.966**	**0.967**	0.986	**0.966**	0.966	0.970
EfficientNet B3	**0.966**	**0.967**	0.995	**0.966**	0.966	0.972

Highest values are highlighted in bold. MCC: Matthew’s correlation coefficient; AUC: area under the curve.

Since the EfficientNet B3 model achieved the highest per-slice test AvgScore and its per-mouse test performance was comparable with other top-performing models, it was selected for toxicity and explainability analysis.

### Early toxicity correlations

**Figure 2:**
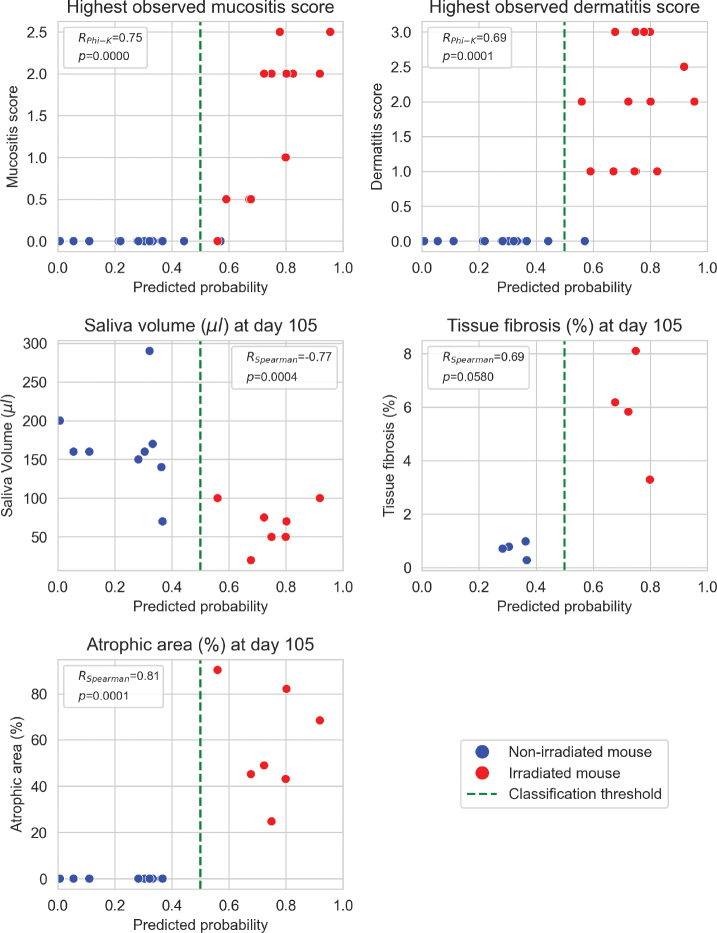
Relationships between model-predicted probability of the irradiated class and toxicity endpoints. Spearman’s (R_Spearman_) and Phi-K's (R_Phi-K_) correlation coefficients are given with p-values.

All investigated toxicity endpoints were correlated with the predicted probability, with absolute phi-K and Spearman correlation coefficients exceeding 0.69 (Figure 2). These correlations were statistically significant (*p* < 0.05) for all endpoints except tissue fibrosis. Higher predicted probabilities of the irradiated class were correlated with higher oral mucositis and dermatitis scores, larger fibrotic and atrophic areas and lower saliva production, indicating that the model predictions aligned with observed toxicity outcomes.

### Model explainability results

**Figure 3:**
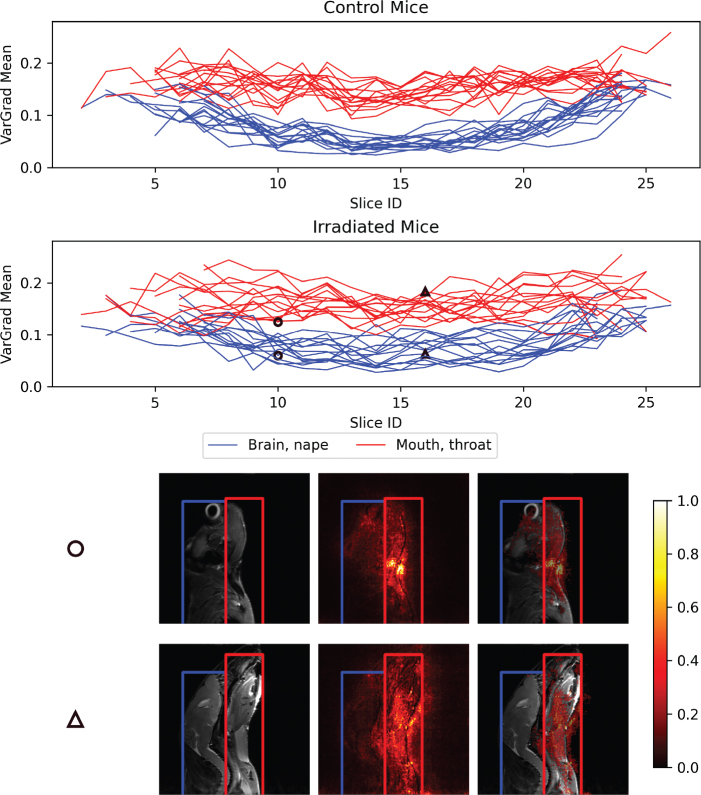
Line plots showing changes in the mean VarGrad values within the brain & nape regions of interest (ROIs; blue) and the mouth & throat ROIs (red) across all image slices for each mouse in the control group (top row) and irradiated group (second row). The two bottom rows display two example T2-weighted (T2w) MR image slices (left), corresponding to the points marked by circles and triangles in the line plots. The corresponding explainability heatmaps, based on normalized VarGrad values (color bar), are shown both as standalone maps (center column) and overlaid on the corresponding T2w slices (right column). Pixels with higher VarGrad values indicate greater contribution to the model’s prediction. Two ROIs are highlighted: non-irradiated areas (brain and nape) in blue, and irradiated areas (mouth and throat) in red. MR: Magnetic Resonance.

**Figure 4:**
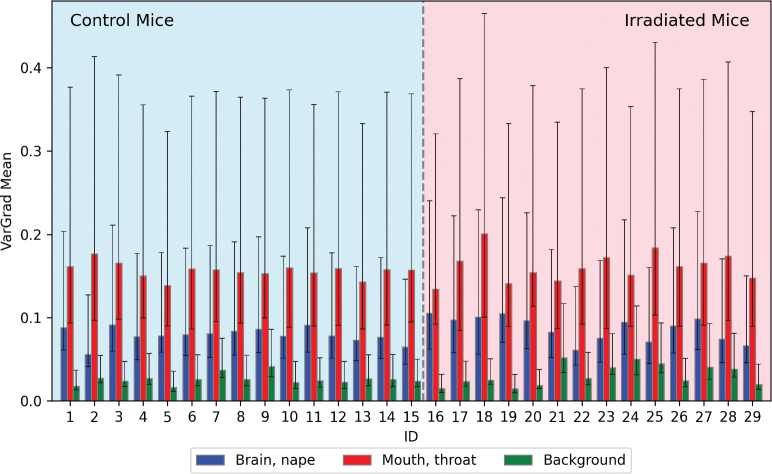
Mean VarGrad values for each mouse across three regions of interest: brain & nape (blue), mouth & throat (red) and background (green). Error bars represent the 25th and 75th percentiles. Statistical analysis using the Mann–Whitney U test confirmed significant differences in VarGrad values between the three regions (p < 0.00001), indicating clear distinctions in model prediction contribution across anatomical areas within the MR images. MR: Magnetic Resonance.

VarGrad heatmaps showed that voxels within the mouth and throat (irradiated region) contributed more to the model predictions than non-irradiated regions (brain and nape), with substantially higher VarGrad values across most MR slices, particularly in the central slices (Figure 3). Explainability heatmaps (Figure 3) also showed that irradiated areas were clearly highlighted, indicating stronger contribution to model predictions than non-irradiated areas. The mean VarGrad values (Figure 4) within the irradiated regions were found to be about twice those within the corresponding non-irradiated regions. However, the difference in VarGrad values between these regions (i.e. mean VarGrad mouth & throat minus mean VarGrad brain & nape) did not vary significantly across treatment groups (*p* = 0.57). Both regions had substantially larger mean VarGrad values than the background. Mann–Whitney U tests confirmed significant differences in VarGrad values between these three regions (*p* < 0.00001).

## Discussion and conclusion

This study demonstrates that CNN models can distinguish between control and irradiated mice using MR images acquired 3–5 days post-irradiation. The best-performing model achieved a per-slice test ACC of 83% and correctly classified 28 out of 29 mice, indicating that subtle radiation-induced tissue changes, undetectable to the human eye but captured in T2w images, can be identified by deep learning. Furthermore, the model’s predicted class probabilities showed strong correlations with two early (8–35 days post-irradiation) and three late (105 days) toxicity endpoints, where higher probabilities of the irradiated class were associated with more severe toxicities. These findings indicate that the model’s predictions reflect tissue toxicity, suggesting that early post-irradiation MR images combined with deep learning may support future toxicity risk assessment. Explainability analyses further reinforced the model’s validity, as importance heatmaps revealed that the CNNs predominantly focused on irradiated regions, with VarGrad values consistently higher in these areas compared to non-irradiated tissue. This pattern suggests that the models learned biologically meaningful image features rather than relying on spurious correlations.

This work builds upon a previous study [17] that applied vision transformers to classify control and irradiated mice using the same dataset. While that study demonstrated the feasibility of deep learning for this task, our approach extends these findings in several important ways. First, CNN-based models achieved markedly higher performance, improving per-slice ACC from 69 to 83%. This improvement was partly driven by aggregating (ensembling) predictions from multiple CV-trained models, which is known to enhance robustness [37] and contributed to a gain from 77 to 83% ACC (Supplementary Table D2, EfficientNet B3). Second, the present study introduced a refined preprocessing pipeline that enhanced image contrast, potentially mitigating noise and outlier voxel values in the 3D MR images. Third, we implemented an automated, criterion-based slice selection process to exclude slices with minimal mouse tissue, reducing the risk of confounding effects. Fourth and finally, beyond classification ACC, this work provides novel insights by correlating model predictions with multiple toxicity scores, including two early and three late endpoints, rather than focusing solely on salivary production. Together, the advances in this study emphasize the added value of CNN-based approaches and the potential clinical relevance of early post-irradiation MRI combined with deep learning.

Various artificial intelligence models have been developed to predict radiation-induced toxicity [4, 38, 39]. These models usually incorporate clinical information, medical imaging such as CT, MR or PET, and dosimetry data such as dose maps or DVH parameters, using either radiomics-based features or direct CNN-based models [9, 40]. For example, Sheikh et al. [41] demonstrated that radiomics features of the salivary gland extracted from MR images could reflect baseline salivary gland function and help predict xerostomia risk following RT in HNC patients. Similarly, van Dijk et al. [42] found that biomarkers from MR images were positively correlated with xerostomia 12 months after RT. Khajetash et al. [9], by employing an ensemble approach using CT and MR images, reported that pre-treatment T1-weighted MR images were better predictors of xerostomia than T2-weighted ones. Nevertheless, these results were based on pre-treatment data, disregarding the variation of patients’ responses to RT.

A few studies have explored changes in medical images during treatment. van Dijk et al. [43] showed that weekly changes in the parotid glands captured by CT images could improve prediction of moderate to severe xerostomia using delta-radiomics features. Liu et al. [44] also used CT radiomic changes in the parotid glands during treatment (at the 10th, 20th and 30th fractions) and 3 months post-radiation to predict acute xerostomia. These studies showed the potential of using post-irradiation image changes to improve toxicity prediction. However, in contrast to our study, they relied on manual contouring of the region of interest for radiomics data extraction, which can be time-consuming and subject to inter-observer variability. Kapoor et al. [45] used CNN models to predict pneumonitis in lung cancer using pre-treatment CT, 3- and 6-month follow-up CT and 3D dose maps, demonstrating the predictive power of the CNN models. In general, there is a lack of studies (and approved markers) for predicting toxicity at an early stage, particularly during or immediately after RT. Future work may include integrating the CNN models with multi-modality inputs, such as dose maps, patient demographics and treatment planning data, to improve predictive performance. Combining these multi-modality inputs with advanced explainability techniques could help highlight tissues or organs in medical images that are at high risk of RT -induced toxicity, further supporting clinical decision-making.

The most apparent limitation of this study is the small sample size (29 mice), which required dividing the data into smaller units, sagittal slices, for model development and analysis. As a result, 2D CNN models were used instead of 3D models, despite the 3D structure of the MR images. However, the use of 2D models allowed for the application of transfer learning with pretrained models, which may have improved model performance given the limited dataset. Another limitation is that only a single explainability method (VarGrad) was investigated. Further investigation of additional model explainability techniques such as LIME [46] or SHAP [47] could provide different insights and comprehensive understanding of model behavior. Furthermore, the irradiated and non-irradiated regions were only approximated using semi-automatic methods. Utilizing available foundation models, such as ‘Segment Anything’ [48], to contour anatomical structures within the 3D MR images [49] could enable more accurate regional quantification of model explainability approaches. Finally, the radiation dose used in this study was relatively high (65 Gy in 10 fractions), which may have caused more tissue damage compared to standard fractionation schemes over longer intervals, and therefore making it more detectable by CNN models. Future studies should explore the sensitivity of these models to lower, clinically relevant doses given at longer intervals.

In conclusion, this study demonstrates the potential of CNN models to detect radiation-induced changes to normal tissue in early post-RT MR images. Strong correlations were found between model predictions and early and late toxicity endpoints, suggesting that early post-therapy MRI features could serve as a proxy for toxicity. These findings support the feasibility of using deep learning on post-treatment medical imaging for early toxicity risk assessment, with implications for future clinical translation.

## Supplementary Material



## Data Availability

The data that support the findings of this study are available from Prof. Eirik Malinen (eirik.malinen@fys.uio.no) but restrictions apply to the availability of these data, which were used under license for the current study, and so are not publicly available.
