# Supplementary material for: CNN-based prediction using early post-radiotherapy MRI as a proxy for toxicity in the murine head and neck
Source: Acta Oncol. 2025 Sep 25;64:44020. doi: 10.2340/1651-226X.2025.44020 (PMC12490106; doi:10.2340/1651-226X.2025.44020)
Supplement: Supplementary file 1 [file AO-64-44020-s1.pdf]

Supplementary material has been published as submitted. It has not been copyedited, or typeset by Acta Oncologica

CNN-based prediction using early post-radiotherapy MRI as a proxy for toxicity in the murine head and neck

Supplementary Materials

Supplementary A. Dataset

Supplementary Table A1. MR slices selected for each mouse after applying the inclusion criteria.

ID	Selected slices	Total
1	2-24	23
2	5-26	22
3	5-24	20
4	4-23	20
5	3-24	22
6	5-25	21
7	6-25	20
8	6-25	20
9	5-26	22
10	6-24	19
11	5-24	20
12	7-24	18
13	6-24	19
14	6-25	20
15	7-24	18
16	6-23	18
17	3-23	21
18	4-24	21
19	3-23	21
20	2-24	23
21	6-25	20
22	5-25	21
23	6-25	20
24	4-23	20
25	7-24	18
26	7-25	19
27	6-26	21
28	7-25	19
29	6-25	20

Supplementary B. CNN model training

Supplementary Figure B1. Full workflow of the CNN model development, from image preprocessing (1), slice selection (2 & 3), model training (4) and testing (5), to model explainability analysis (6).

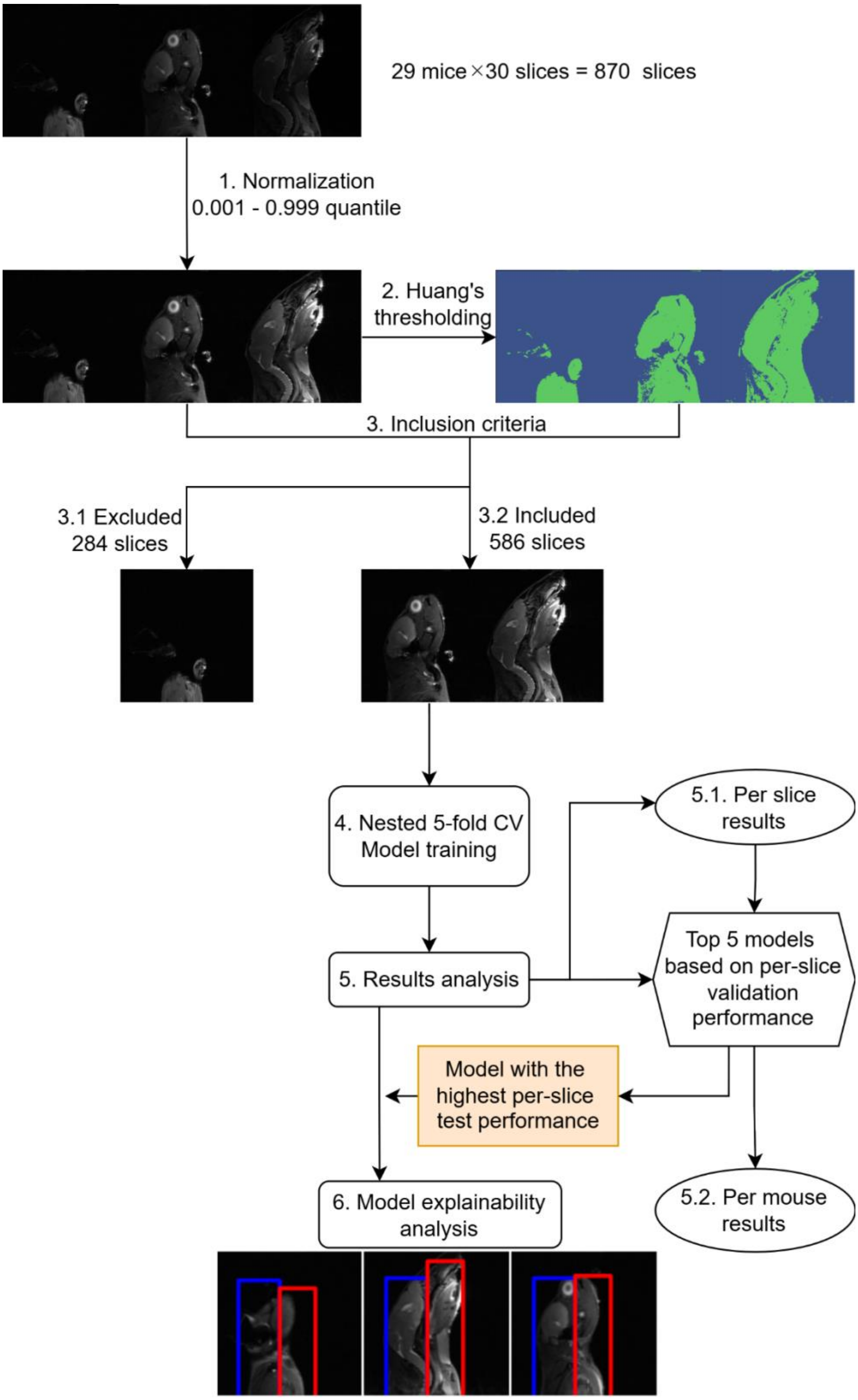

Supplementary Figure B2. The nested cross-validation scheme for training, validating, and testing CNN models. The dataset was divided into five folds (top), with three folds (green) used for training, one fold (orange) used for validation, and one fold (red) used for testing in each run, giving 20 runs in the scheme. Each MR slice was used as test data in four independent runs, resulting in four different predicted class probabilities P_{si} for $i = 1, 2, 3, 4$ runs per slice. The final test predictions $\bar{P}_S$ were obtained based on ensemble averaging of these probabilities where $\bar{P}_S = \frac{1}{4}\sum_{i=1}^{4} P_{si}$. All predictions in the figure refer to the predicted class probabilities.

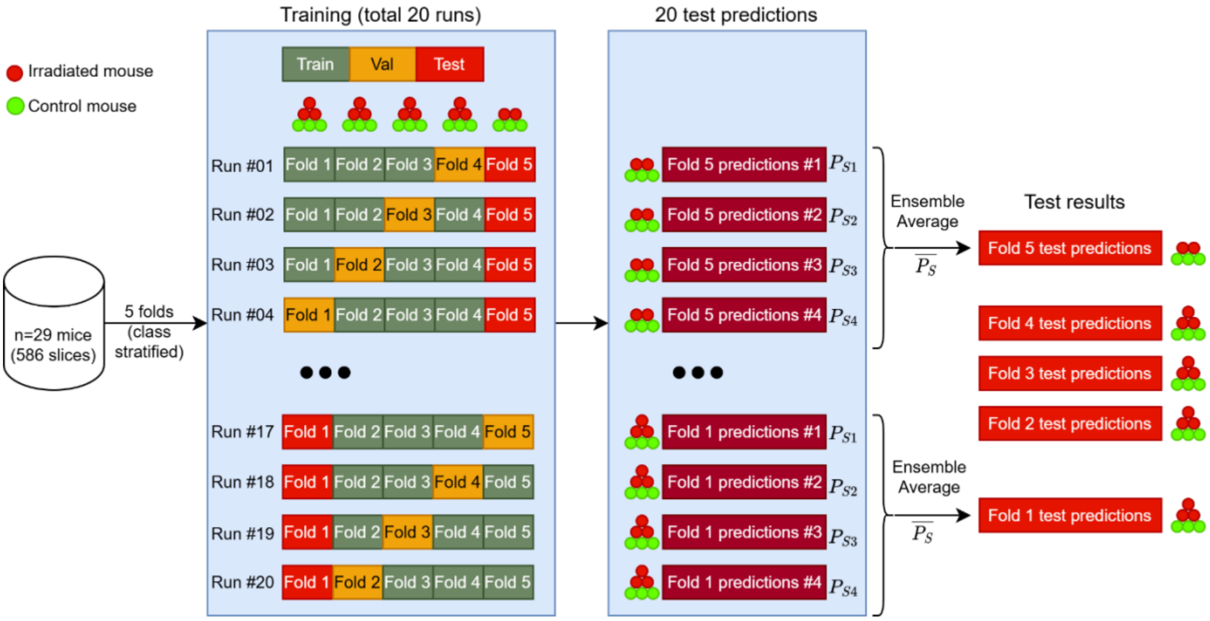

Supplementary C. Performance metrics

In the present study, five different performance metrics were used to evaluate the performance of the CNN models: Accuracy, F1 Score Irradiated, F1 Score Control, Matthew's correlation coefficient (MCC), and area under the receiver operating characteristic curve (AUC) [1].

The first four metrics are based on the number of true positives (TP), false negatives (FN), true negatives (TN), and false positives (FP) from the confusion matrix, using a decision threshold of 0.5 for the prediction probability. The basic metric is accuracy (ACC), which is the proportion of correctly classified positive and negative cases, as:

$$\mathrm{ACC} = \frac{\mathrm{TP} + \mathrm{TN}}{\mathrm{TP} + \mathrm{FN} + \mathrm{TN} + \mathrm{FP}} .$$

We also report the F1 Score on irradiated and control cases, defined as:

$$\mathrm{F1\ irradiated} = \frac{2\mathrm{TP}}{2\mathrm{TP} + \mathrm{FN} + \mathrm{FP}} ,$$

$$\mathrm{F1\ control} = \frac{2\mathrm{TN}}{2\mathrm{TN} + \mathrm{FN} + \mathrm{FP}} .$$

We also include the MCC [1], which is defined as:

$$\mathrm{MCC} = \frac{\mathrm{TP} \times \mathrm{TN} - \mathrm{FP} \times \mathrm{FN}}{\sqrt{(\mathrm{TP} + \mathrm{FP})(\mathrm{TP} + \mathrm{FN})(\mathrm{TN} + \mathrm{FP})(\mathrm{TN} + \mathrm{FN}}} .$$

As the MCC ranges from -1 to 1 while all other metrics used in the present study range from 0 to 1, we rescaled MCC to the interval 0 to 1 for easier comparison, as follows:

$$\mathrm{Scaled\ MCC} = \frac{\mathrm{MCC} + 1}{2} .$$

We included the AUC, which is another metric ranging from 0 to 1 where 1 is perfect prediction, 0.5 is random prediction, and 0 is reversed prediction. AUC measures the area under the receiver operating characteristic curve (ROC) [1], which is produced by tracing the true positive rate (TPR, also referred to as sensitivity) as a function of the false positive rate (FPR) when varying the decision threshold from 0 to 1. TPR and FPR are given as:

$$\mathrm{TPR} = \frac{\mathrm{TP}}{\mathrm{TP+FP}} , \qquad \mathrm{FPR} = \frac{\mathrm{FP}}{\mathrm{FP+TN}} .$$

Finally, the AvgScore was calculated by averaging the five defined performance metrics:

$$AvgScore = \frac{\mathrm{ACC} + \mathrm{AUC} + \mathrm{Scaled\ MCC} + \mathrm{F1\ control} + \mathrm{F1\ irradiated}}{5}$$

Supplementary D. Model performances

Supplementary Table D1. Per-slice performance metrics of all 17 CNN models on the validation set, sorted in descending order of AvgScore. The highest value of each metric is given in **bold**.

Model	ACC	Scaled MCC	AUC	F1 Irradiated	F1 Control	AvgScore
EfficientNet B1	**0.823**	**0.827**	0.892	0.808	**0.834**	**0.837**
EfficientNet B6	0.818	0.819	**0.898**	0.802	0.827	0.833
EfficientNet B0	0.816	0.818	0.894	0.804	0.820	0.831
EfficientNet S	0.816	0.818	0.890	0.799	0.828	0.830
EfficientNet B3	0.817	0.819	0.883	0.807	0.820	0.829
EfficientNet B7	0.813	0.819	0.888	**0.812**	0.807	0.828
EfficientNet M	0.815	0.817	0.875	0.802	0.824	0.827
EfficientNet B4	0.809	0.816	0.883	0.798	0.815	0.824
EfficientNet B2	0.807	0.812	0.882	0.800	0.810	0.822
ResNet101	0.808	0.811	0.879	0.791	0.819	0.821
EfficientNet B5	0.807	0.812	0.882	0.798	0.807	0.821
Inception	0.796	0.798	0.872	0.780	0.803	0.810
ResNet152	0.795	0.798	0.871	0.778	0.804	0.809
ResNet50	0.791	0.795	0.856	0.776	0.796	0.803
MobileNet	0.736	0.748	0.829	0.698	0.744	0.751
VGG16	0.541	0.544	0.629	0.291	0.587	0.518
VGG19	0.523	0.517	0.610	0.173	0.591	0.483

Supplementary Table D2. Average per-slice (top) and per-mouse (bottom) validation performance, average test performance for the 20 runs, and ensemble test results for the top 5 performing CNN models on the validation set.

Per-slice results							
Model		Accuracy	Scaled MCC	AUC	F1 irradiated	F1 control	AvgScore
Validation							
EfficientNet	B1	**0.823**	**0.827**	0.892	**0.808**	**0.834**	**0.837**
EfficientNet	B6	0.818	0.819	**0.898**	0.802	0.827	0.833
EfficientNet	B0	0.816	0.818	0.894	0.804	0.820	0.831
EfficientNet	S	0.816	0.818	0.890	0.799	0.828	0.830
EfficientNet	B3	0.817	0.819	0.883	0.807	0.820	0.829
Test prior to ensembling							
EfficientNet	B1	0.731	0.736	0.819	0.710	0.744	0.748
EfficientNet	B6	0.731	0.738	0.831	0.715	0.738	0.750
EfficientNet	B0	0.744	0.753	0.844	0.738	0.740	0.764
EfficientNet	S	0.757	0.762	0.842	0.744	0.765	0.774
EfficientNet	B3	**0.766**	**0.769**	**0.864**	**0.752**	**0.770**	**0.785**
Test							
EfficientNet	B1	0.778	0.778	0.870	0.760	0.794	0.796
EfficientNet	B6	0.794	0.793	0.886	0.783	0.803	0.812
EfficientNet	B0	0.805	0.807	0.888	0.807	0.804	0.822
EfficientNet	S	0.783	0.783	0.889	0.771	0.794	0.804
EfficientNet	B3	**0.833**	**0.834**	**0.914**	**0.832**	**0.834**	**0.849**

Per-mouse results							
Model		Accuracy	Scaled MCC	AUC	F1 irradiated	F1 control	AvgScore
Validation							
EfficientNet	B1	0.940	0.946	0.972	0.939	0.939	0.947
EfficientNet	B6	**0.957**	**0.961**	**0.994**	**0.946**	**0.961**	**0.964**
EfficientNet	B0	0.948	0.954	0.983	0.945	0.946	0.955
EfficientNet	S	0.942	0.947	0.983	0.936	0.945	0.951
EfficientNet	B3	0.933	0.940	0.989	0.930	0.931	0.945
Test prior to ensembling							
EfficientNet	B1	0.833	0.850	0.917	0.801	0.845	0.849
EfficientNet	B6	0.837	0.861	0.939	0.827	0.825	0.858
EfficientNet	B0	0.828	0.853	0.936	0.827	0.811	0.851
EfficientNet	S	0.860	0.872	0.942	0.860	0.850	0.877
EfficientNet	B3	**0.900**	**0.909**	**0.972**	**0.896**	**0.897**	**0.915**
Test							
EfficientNet	B1	0.897	0.897	0.981	0.889	0.903	0.913
EfficientNet	B6	**0.966**	**0.967**	**1.000**	0.963	**0.968**	0.973
EfficientNet	B0	0.931	0.935	0.971	0.933	0.929	0.940
EfficientNet	S	0.966	0.967	0.986	**0.966**	0.966	0.970
EfficientNet	B3	**0.966**	**0.967**	0.995	**0.966**	0.966	**0.972**

Reference

1. Chicco D, Jurman G. The Matthews correlation coefficient (MCC) should replace the ROC AUC as the standard metric for assessing binary classification. Biodata Min. 2023;16(1).
